# Cervical adenocarcinoma presenting as a cardiac tamponade in a 57-year-old woman: a case report

**DOI:** 10.1186/1752-1947-5-594

**Published:** 2011-12-21

**Authors:** Elie Azria, Marion Dufeu, Pedro Fernandez, Francine Walker, Dominique Luton

**Affiliations:** 1 Department of Gynecology Obstetric, Bichat Claude Bernard Hospital, 46 Rue Henri Huchard, 75877 Paris Cedex 18, France; 2 Paris 7 Diderot University, Paris, France; 3 Department of Radiology, Bichat Claude Bernard Hospital, 46 Rue Henri Huchard, 75877 Paris Cedex 18, France; 4 Department of Pathology, Bichat Claude Bernard Hospital, 46 Rue Henri Huchard, 75877 Paris Cedex 18, France

## Abstract

**Introduction:**

Pericardial effusion as a complication of malignant gynecological disorders is rare. Few cases of endometrial cancer, squamous cell carcinoma of the cervix, ovarian cancer and uterine carcinosarcoma have been previously reported. We report the first case of cardiac tamponade secondary to a cervical adenocarcinoma.

**Case presentation:**

A 54-year-old Caucasian woman, without any relevant medical history and no gynecological aftercare, was admitted to our hospital emergency room with severe dyspnea. Echocardiography revealed severe pericardial effusion with a swinging heart. An emergency pericardial drainage was performed through a pericardial window, which permitted the draining of 700 mL of bloody fluid and a pericardial biopsy. Cytological examination of the fluid revealed atypical cells, and the biopsy specimen showed tumor emboli suggestive of adenocarcinoma. Magnetic resonance imaging showed a 35 mm cervical lesion indicative of an endocervical tumor. Exploratory laparoscopy revealed diffuse peritoneal lesions and histological examination of cervical curettage showed a poorly differentiated micropapillary adenocarcinoma of the cervix.

**Conclusion:**

Carcinomatous pericarditis as the first symptom of a malignant gynecological adenocarcinoma has not, to the best of our knowledge, been documented before. This case highlights the extreme severity of pericardial effusion secondary to cervical adenocarcinoma, a sign of advanced disease. Gynecological malignancies have to be considered in cases of neoplastic pericardial effusion.

## Introduction

Cardiac tamponade as a complication of a malignant gynecological disorder is a very rare occurrence. Very few cases of malignant pericardial effusion due to endometrial cancer [1-4], squamous cell carcinoma of the cervix [5-9], ovarian cancer [10] or uterine carcinosarcoma [11] have been reported. We report a case of cervical adenocarcinoma revealed by symptoms of cardiac tamponade.

## Case presentation

A 54-year-old Caucasian woman, without any relevant medical history and no gynecological aftercare, was admitted to our hospital emergency room with dyspnea that had progressed over 10 days and was worsening. Upon admission, we noted blood oxygen saturation of 89%, tachycardia (125 beats per minute) and hyperthermia (38.9°C). Her blood pressure was 120/80 mmHg. Electrocardiography showed sinus tachycardia and echocardiography revealed severe pericardial effusion with a swinging heart. An emergency subxiphoid incision with pericardial drainage was performed through a pericardial window, which permitted draining of 700 mL of bloody fluid and a pericardial biopsy. Cytological examination of the fluid revealed atypical cells, and the biopsy specimen showed tumor emboli suggestive of adenocarcinoma. Immunostaining indicated elevated levels of tumor marker, cytokeratin 7 (CK7). Her blood concentrations of carbohydrate antigen (CA) 125 and CA 15.3 were 4667 IU/L and 451 IU/L, respectively, suggesting ovarian malignancy.

**Figure 1:**
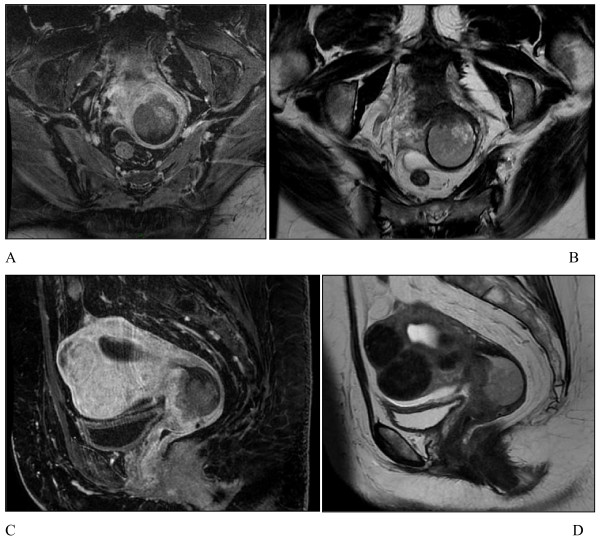
**MRI findings**. Mid-sagittal pelvic **(A) **T1-weighted post gadolinium and **(B) **T2-weighted images, showing an endocervical tumor with necrotic areas and large typical uterine interstitials fibroids. Axial pelvic **(C) **T1-weighted post gadolinium and **(D) **T2-weighted images at the tumor level.

**Figure 2:**
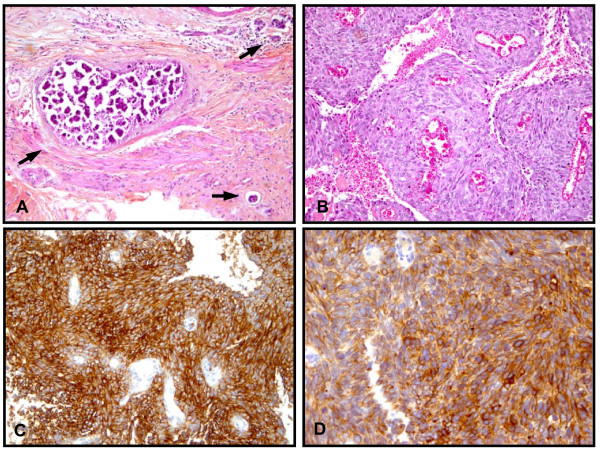
**Pathology findings**. **(A) **Pericardial tissue showing intralymphatic dissemination of the endocervical carcinoma with papillary pattern (arrows), hematoxylin and eosin stain (× 100). **(B) **Undifferentiated area of endocervical adenocarcinoma, hematoxylin and eosin stain (× 100). **(C) **Undifferentiated area of endocervical adenocarcinoma showing a high immunoreactivity with anti-CA 125 antibody (× 100). **(D) **Undifferentiated area of endocervical adenocarcinoma. Anti-CK7 antibody shows the same high immunoreactivity as the pericardial metastasis (× 200).

When stabilized, our patient was transferred to our department for further investigations. A gynecological examination only found a large uterus. A physical examination of her cervix was normal. An ultrasound examination showed uterine myomatosis, but no endometrial abnormality. Her ovaries were not seen. Magnetic resonance imaging (MRI) showed a 35 mm cervical lesion indicative of an endocervical tumor (Figure 1). A thoracic tomodensitometry and liver ultrasound were normal. Exploratory laparoscopy found normal ovaries, with no ascites, but diffuse peritoneal lesions suggestive of carcinosis. A bilateral adnexectomy and multiple peritoneal biopsies were performed. Endocervical curettage revealed necrotic tumor tissue. Cervical biopsies were performed. Histological examination showed a poorly differentiated micropapillary adenocarcinoma of her cervix with peritoneal and ovarian involvement (Figure 2). Immunostaining showed that the tumor cells were strongly positive for CA 125, Kit-ligand-1, Ki67, and CK7. Three weeks after this surgical procedure, intravenous chemotherapy with carboplatin and paclitaxel was initiated. Our patient's progression was unfavorable; three days after the first course of chemotherapy she was admitted to our intensive care unit and given thrombolytic therapy for severe pulmonary embolism with no sign of pericardial effusion recurrence. She died due to heart failure after three days in a context of severe pulmonary arterial hypertension.

## Discussion

Malignant neoplasia from any organ can potentially metastasize to the pericardium [12], but there are few data concerning pericardial metastasis of gynecological cancer and most are regarding ovarian carcinoma [13]. In our review of the literature, we identified four reported cases of endometrial adenocarcinoma metastasizing to the pericardium, diagnosed antemortem [1-4], but none of cervical adenocarcinoma. Pericardial involvement is usually silent clinically and is often noticed only on postmortem examination [12], but it can also cause effusion and, rarely, tamponade [1,2]. In an autopsy series of 11,432 patients reported by Butany *et al. *[14], a total of 264 patients were found with cardiac metastases. The primary neoplasm was gynecological in only two (< 1%) of these cases. The cardiac tissue involved was pericardium in most cases. The pericardium is rarely the only location of metastatic spread from the primary tumor [15], and is suggestive of disseminated disease. In our case, it was for this reason and because of the immunostaining appearance of the pericardial biopsy, that exploratory laparoscopy was performed, which showed peritoneal carcinosis.

## Conclusion

When symptomatic, pericardial metastases tend to occur as secondary manifestations of the cancer [2,3,6,11]. Carcinomatous pericarditis as the first symptom of a malignant gynecological adenocarcinoma has not, to our knowledge, been documented before.

Where medium-term follow-up has been reported, the outcome of patients with endometrium adenocarcinoma with pericardial effusion was progression to death in two of the four reported cases [1,3]. In the two other cases, patients were alive at six-month follow-up [2,4]. For our patient, the interval between tamponade and death was very short (33 days).

According to our findings and previous reports, cardiac tamponade can be considered as indicative of a very poor prognosis when it complicates the progression of a cervical or endometrial adenocarcinoma.

This case highlights the extreme severity of pericardial effusion secondary to cervical adenocarcinoma, a sign of advanced disease. Gynecological malignancies have to be considered in cases of neoplastic pericardial effusion.

## Consent

Written informed consent for publication could not be obtained from any immediate relatives despite all reasonable attempts. Every effort has been made to protect the identity of our patient and there is no reason to believe that any of her immediate relatives would object to publication.

## Competing interests

The authors declare that they have no competing interests.

## Authors' contributions

EA and MD wrote the manuscript. PF analyzed and interpreted the imaging of the patient and was a contributor in writing the manuscript. FW performed the histological examination and was a major contributor in writing the manuscript. DL critically reviewed the manuscript and provided significant help in writing. All authors have read and approved the final version of the manuscript.
